# Adaptive Sensing Based on Profiles for Sensor Systems

**DOI:** 10.3390/s91108422

**Published:** 2009-10-26

**Authors:** Yoshiteru Ishida, Masahiro Tokumitsu

**Affiliations:** Department of Knowledge-Based Information Engineering, Toyohashi University of Technology / 1-1, Tempaku, Toyohashi, Aichi 441-8580, Japan; E-Mail: tokumitsu@sys.tutkie.tut.ac.jp

**Keywords:** sensor systems, profiles, adaptive sensing, dynamic relational network

## Abstract

This paper proposes a profile-based sensing framework for adaptive sensor systems based on *models* that relate possibly heterogeneous sensor data and *profiles* generated by the models to detect events. With these concepts, three phases for building the sensor systems are extracted from two examples: a combustion control sensor system for an automobile engine, and a sensor system for home security. The three phases are: modeling, profiling, and managing trade-offs. Designing and building a sensor system involves mapping the signals to a model to achieve a given mission.

## 1. Introduction

Sensor fusion involves integrating multiple and heterogeneous sensors, intelligent sensors to integrate detection and post-processing of signals, and sensor networks of simple low-power sensors. Furthermore, sensor systems based on profiles that adapt to the environment require not only domain-dependent sensor technology on which each sensor depends but also domain-independent common frameworks. However, such frameworks are lacking, so this paper proposes a basis for such frameworks.

Various sensor technologies and sensors for detecting environmental properties have been developed, ranging from low-cost, low-fidelity sensors to expensive sensors with high fidelity. Meanwhile, rapid progress in wireless technology and information networks now allows many distributed sensors to be aggregated and organized [1-4], ranging in extent from within one room to entire buildings and production plants. Innovation in sensor networks has been driven by the Internet and breakthroughs in ubiquitous computing and even “pervasive computing” [5].

With the advent of low-power sensors and networking technology, sensor networks have been attracting increasing attention. However, for these to be useful, it is necessary to synthesize the large quantities of data collected from such sensor networks. We will use the term *sensor systems* in a broader sense than usual to encompass systems that can be extrapolated and interpolated in the dimensions of time, space and event. Sensing may be formalized as a mapping from the properties of objects to a model for a specific mission. As clarified in Section 2.2, modeling relates many measurements (possibly multi-modal) at each point of the multi-dimensional time series, whereas profiling allows designing in the event dimension. Finally, adaptation involves organizing the properties at different time points (past, current, future) and over different time spans.

Similarly to the difference between machine computation and human inference, artificial sensing tends to focus on numerical precision while biological sensing focuses on constructing a model for a specific mission: survival of the individual animal. Thus, biological sensing is more goal-oriented than artificial sensing, which merely measures or detects a property of the target object. Another remarkable difference is that biological sensing can deal with a collection of properties of qualitatively different types, while artificial sensing mainly handles a collection of properties of the same type. This comes from the goal-oriented nature of biological systems that must deal with noisy, non uniform, and uneven data. We will explain the design principle and basic concepts required for adaptive sensing using two examples: automobile engine monitoring and home security with adaptive sensor systems.

## 2. Concepts

Considering the future direction of sensor systems and available technologies, we focus on the following three factors in the process of designing sensor systems:

- Modeling allowing heterogeneous data: heterogeneous data, not only in the sense of distinct but related data such as temperature, pressure, flow, but also distinct in time scale, sampling period, and level of noise
- Profiling to minimize the rate of false-alarms and missed-alarms
- Tuning trade-offs while considering adaptation

These three factors are closely related and cannot be considered separately. When designing a sensor system for home security, for example, it is necessary to adapt to both the varying lifestyles of residents, as well as seasonal or daily climate changes, and the profiles should reflect these variations. Thus, when designing a sensor system to monitor environmental conditions, modeling may be needed to relate direct physical measurements reflecting the natural conditions with measurements reflecting human activities, for example.

Sensing may be divided into two processes: identifying or measuring properties of target objects, and organizing sensing to build a model for a mission. The former is data-driven, while the latter tends to be goal-driven. Expressed differently, the former processes direct signals such as in visual and auditory perception, while the latter organizes collected and processed signals as in the nervous system and the brain. Also, some senses such as smell, taste and touch may be difficult to classify into the former or the latter. This difficulty arises because the properties of target objects corresponding to these senses are complex, rather than being a simple one-dimensional line such as measuring temperature. Before examining the three factors for sensor systems, we should first extend and formalize measurement and modeling, which two important processes of sensor systems.

### 2.1. Measurement

As a process, some properties of objects should be measured before detection, identification, and prediction of events (which are the task of the model). In a simple case, measurement can be considered to be mapping to a one-dimensional line a property of the target object such as temperature, pressure, mass, or any other property whose intensity may be mapped onto a one-dimensional line. Although complex measurements are interesting and it is tempting to formalize them by using a mathematical concept of *manifold*, we leave this pre-process before modeling for future studies. Here, we focus on profiling, which is a post-process after modeling. Profiles, which are sets of features that characterize events of interest and are used for detecting, identifying, and predicting the events, may be compiled from multiple local orderings.

Regarding the taxonomy of measurement, there are two main approaches: absolute and relative measurement. When weighing an object, absolute measurement uses a conventional scale that maps the force of gravity (proportional to the mass) to a point in a one-dimensional line, while relative measurement uses a balance and several weights to which the target object is compared. Another example is measuring temperature: absolute measurement typically uses a conventional mercury thermometer, while the Galileo thermometer measures temperature in a relative manner with several bulbs indicating whether the current temperature is above or below the temperature of each bulb. For sensor systems, relative measurements should also be considered because of flexibility (such as robustness against measurement noise, *gracefully degrading* against device failure, and availability for different uses), although precision may be lacking.

### 2.2. Modeling

If sensing is divided into two processes, measurement and post-processing of measured data (i.e., detection identification, and prediction), then conventional sensing is biased toward measurement. However, when a sensor system is based on heterogeneous multiple sensors, it will be biased toward post-processing.

This intelligent information processing may be done purely in a data-driven manner, but it can also be done by a model reflecting causal and heuristic relations within the target objects. We focus on the information processing involving *models* for detection, identification and even prediction of events, and use a dynamic relational network [6,7] as the model.

By fully exploiting the goal-driven mechanism, intelligent sensing improves flexibility, for the goal-driven mechanism can deal with unexpected situations without enumerating all possible cases. This flexibility is essential for sensor systems because the goal-driven activation of sensors can save energy and can guide the system to focus on the target. However, flexibility involves a trade-off, since it can lead to unexpected and undesired actions, such as in the frame problem discussed in artificial intelligence.

The dynamic relational network may be considered as an extension of relative measurement, and recognizes that much information is contained not only in each sensor value but also in the relation (and its dynamics) among sensor values. Even when a target object is monitored by multiple homogeneous sensors, the relation among sensor values may be used to identify abnormal sensors if redundancy is sufficient. If there are multiple heterogeneous sensors, their relation and the dynamics can be used as a basis for profiles that characterize the target events.

Sensors, and hence sensor data, will not be independent if they focus on common objects or the same events; they will be mutually dependent and interrelated. The relations may be physical or experimental, deterministic or probabilistic. Using the information embedded in each sensor as well as that embedded in the relation among sensors is at the heart of the dynamic relational network. Statistical correlation has been used, for example, for the sensors for combustion control systems (Section 4) and for the sensors for home security (Section 5). Even when heterogeneous relations are involved in a network, the relations could be involved if at least the condition specifying when the relation holds (or equivalent condition when the relation does not hold) is defined.

## 3. Design Principles of Sensor Systems

### 3.1. Modeling

As stated above, one role of the model is to relate multiple sensor data that are possibly heterogeneous to map a collection of sensor data to a higher function of detection, identification, and prediction of events. This will be done by evaluating the credibility of sensor values taking into account consistency of the current sensor data with the relations among sensors [6]. An important function of the dynamic relational network is that it involves evaluating the sensor data based on consistency with other sensor data within the sensor system. As the data changes dynamically, the consistency also changes, and hence the evaluations as well. The evaluation is done for each sensor by assigning a continuous value (called credibility) ranging from 0 (not credible) to 1 (fully credible). There may be many models and algorithms for obtaining the credibility other than those proposed [7].

Another role of the model is to define and generate profiles in several ways. In the combustion control sensor system, for example, the substructure (of the network expressing relations among sensors) and model parameters (such as threshold to determine whether the sensor data are consistent with the relations) are used as profiles. In the home security sensor system, profiles characterizing the resident's behavior are accumulated as parameters of a Markov model that reflects the pattern dynamics of sensor activities.

### 3.2. Profiling

Since this paper attempts to realize adaptive sensing by organizing profiles, profiling plays an important role in the sensor system. Profiling has been widely studied and used even when restricted to humans. For example, DNA profiling is commonly used to identify evidence and to narrow down the scope of suspects in criminal cases.

In the home security example, we focus on profiling the activities and behavior of humans (residents) in their daily life, particularly in their homes. Profiles of a resident are used to detect anomalies in their daily life such as housebreaking by an intruder, a collapse or loss of mobility due to sudden illness (e.g., heart attack), and long absence due to wandering caused by an illness (e.g., Alzheimer's disease). In this paper, we deal with the first two: housebreaking and collapsing.

Since the mission of sensor systems is detection of events, we define profiles as information that can be used to detect events. Profiles may be hierarchically structured from specific events to an intermediate category of a certain collection of events, or events up to the most general kind of abnormal event (and its complement, the normal event). Since the taxonomy of profiles is out of the scope of this paper, we will only use the above three: specific event level, event category level, and the most general abnormal/normal level. In the example of the combustion engine sensor system, the specific event level is sensor faults and process anomalies such as abnormal air flow; the event category level is sensor faults and process failures in cruise mode. Profiles for the event level are parameters of the model, and those for the event category level are the network structure and substructure.

By definition, each profile has a corresponding target. In the context of this paper, the target is a specific event. If the profile information is divided into two parts, an event-specific part and an event common part, the former is more important. As explored in the examples below, profiles depend on the environment and on the situation where the sensor system is installed. Thus, profiles must be customized based on the environment and situation. Adaptation allows the profiles to absorb the specific information dependent on the environment and situation.

Regarding the relation between profiles and adaptation, we assume that adaptation will occur in the following three cases: when parameters for the model as the basis of defining consistency are updated; when profiles are added, deleted or replaced; and when the thresholds determining alarm conditions are renewed.

### 3.3. Trade-offs

When designing sensor systems, there are at least two trade-offs to be considered: a sensor sensitivity trade-off and a profile character trade-off.

First, sensor sensitivity controls the trade-off between a false-alarm and a missed-alarm. That is, if the sensors are too sensitive, the false-alarm rate will tend to increase, while if the sensors are too insensitive, the missed-alarm rate will tend to increase. False-alarms are when the system issues an alarm even though an anomaly did not occur, whereas missed-alarms are when the system fails to issue an alarm even though an anomaly actually occurred.

Secondly, there is a trade-off in the spectrum of using profiles of normal events and using those of abnormal events. We find that the missed-alarm rate is greatly decreased when the profiles of abnormal events are incorporated in detection for both examples, rather than detecting anomalies by mismatches against the normal profile. For some specific applications such as intrusion detection, even artificially synthesized events (which are virtual in the sense that they are not originated from real abnormal events) could help decrease the missed-alarm rate.

Acquired immunity involves a mechanism of generating diverse profiles for abnormal events (e.g., antibodies). Indeed, synthesizing profiles for abnormal events may be a tough problem even for biological systems, which is perhaps why acquired immunity exists. Profiles of abnormal events and normal events are asymmetric particularly in their availability, for abnormal events are rare and cannot be made to happen. We have discussed information systems learned from the immune system elsewhere [7]. An engineering conjecture worth mentioning here is that involving profiles for abnormal events would improve the trade-off (the ROC curve would be shifted toward the axes). This conjecture has been experimentally tested in several domains including the two examples given below. Another conjecture is that these abnormal events may not necessarily exist. Indeed, we do not have any means to check whether the abnormal events would really occur or not. Thus, profiles for these abnormal events may be synthesized by an appropriate method, such as by recombining already occurred events. There is no distinction between virtual and real events as far as abnormal events are concerned. This is another asymmetry between normal and abnormal events when the system is placed as an open system. However, this conjecture requires extensive tests in many domains.

We could use profiles of both normal events and abnormal events when the situation (real-time restriction, computation complexity and availability of profiles) allows. However, we focus on the problem of how much we can do without resorting to the profiles of abnormal events.

## 4. Combustion Control Sensor Systems Example [6]

In this example, multiple heterogeneous sensors of the combustion control system for an automobile engine are used for identifying events such as anomalies of the system and sensors themselves. Only normal data are used for building the relational network and extracting profiles characterizing the normal data, and only the cruise phase is used for the normal data. In building the dynamic relational network, statistical methods as well as time series analysis is used. Parameters (thresholds) of the network are used as profiles. Building the dynamic relational network and extracting profiles are carried out off-line.

### 4.1. Modeling: Identifying relations among sensors

A dynamic relational network can be built in two main phases:

1. *Relation Addition Phase*: Find causally related sensors by investigating correlations by checking indices such as coefficients of correlation.
2. *Relation Deletion Phase*: Remove those arcs from sensor *A* to *B* if the test from sensor *A* to *B* generates false positives or false negatives.

**Figure 1.**
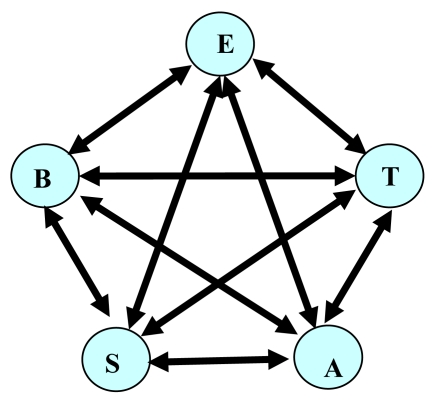
Sensors for a combustion system control (E: Engine revolution speed; B: Battery voltage; T: Throttle position; S: Automobile speed; A: Air flow) have as a statistical correlation with each other. The network turns out to be complete.

Building a dynamic relational network starts from statistical analysis of sensor data. The *Relation Addition Phase* can be done by calculating the coefficient of correlation in statistical analysis. In the *Relation Deletion Phase*, a regression line of sensor data *B* is first expressed with respect to sensor data *A*. The real data of sensor *B* when the target part is non-faulty are then compared with the regression line to check that the regression line does not cause false positives. Also, in the *Relation Deletion Phase*, the real data of sensor *B* when the target part is faulty are compared with the regression line to check that the regression line does not cause missed-alarms. Arcs that cause false-alarms can be removed when only normal data (data when no fault exists) are available, while removal of arcs causing missed-alarms requires abnormal data (data when faults exist). *Sa* indicates the data from sensor *A*. In the *Relation Addition Phase*, arcs between *A* and *B* are added if: | coefficient of correlation between *Sa* and *Sb* | ≥ *θ*. Figure 1 shows a network built when *θ* = 0.4 and only the *Relation Addition Phase* is used.

### 4.2. Profiling: Identifying events and their characteristic parameters and thresholds

Depending on the specifications of the diagnosis, statistical analysis using the coefficient of correlation for the *Relation Addition Phase* and regression analysis for the *Relation Deletion Phase* would suffice for building the network. However, if the time series pattern is critical and a more sophisticated diagnosis is required, time series analysis is needed for the *Relation Addition Phase* (using a mutual correlation matrix) and/or for *Relation Deletion Phase* (prediction by the models of time series analysis). As reported below in the case of the combustion control system for an automobile engine and for a particular fault in an air-flow sensor, a statistical analysis of up to the *Relation Addition Phase* for building the network suffices. However, time series analysis (with VAR model) is used to determine the sign of an arc (evaluation from node *i* to node *j*) in online diagnosis.

**Figure 2.**
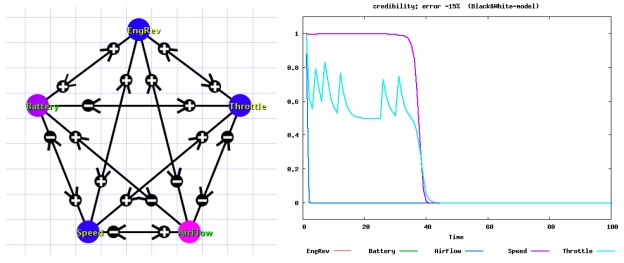
The left network is a snapshot used for trial diagnosis. The sign attached to each arc is a snapshot of evaluation based on the sensor data. The node color indicates credibility: blue nodes correspond to high credibility, and red nodes to low credibility (i.e. evaluated as faulty) (left). The right plot shows diagnosis by the network when the air flow sensor is faulty. The plotted line shows the time evolution of credibility for the sensor; although the credibility for the faulty sensor is evaluated as low (0), those of other sensors are also dragged to 0 (right) [6]. © 2006 Springer.

The signs of arcs in the network change dynamically in online diagnosis; Figure 2 shows only a snapshot of signs. The network structure does not change during the diagnosis. In online diagnosis using the network, the sign of a test from node *i* to node *j* is evaluated online. Let *x*(*t*) and *y*(*t*) be sensor data corresponding to nodes *i* and *j* respectively.

Time series analysis is used for determining thresholds and for deleting relations that are unfavorable for detecting events. As a model for the time series analysis, the vector autoregressive (VAR) model, which is a multivariate extension of the autoregressive (AR) model, is used. In the AR model, the target variable (explained variable) is estimated with respect to its past values (explaining variable). In the VAR model, however, not only its own past values but also those of related variables are involved. Let *x*(*t*) and *y*(*t*) be explained variables; *x*(*t* − 1), …, *x*(*t* − *m*); *y*(*t* − 1), …, *y*(*t* − *m*) be explaining variables; and *a*_1_, …, *a_m_*; *b*_1_, …, *b_m_*; *c*_1_, …, *c_m_*; *d*_1_, …, *d_m_* be autoregressive coefficients. Then, the VAR model of order *m* is expressed as:

$$\begin{array}{l} x(t)=\underline{a_{1}x(t-1)+\ldots +a_{m}x(t-m)+b_{1}y(t-1)+\ldots +b_{m}y(t-m)}+\varepsilon_{x}\\ y(t)=\underline{c_{1}x(t-1)+\ldots +c_{m}x(t-m)+d_{1}y(t-1)+\ldots +d_{m}y(t-m)}+\varepsilon_{y} \end{array}$$

where the underlined parts (*x*′(*t*), *y*′(*t*)) represent predicted values while *ε* are the residual errors. In offline data handling before online diagnosis, autoregressive coefficients and the residual errors between the training data and predicted values *x*′(*t*) are normalized with respect to *x*(*t*). Let these normalized residual errors be *p*′(*t*). In online diagnosis based on the network, tests corresponding to arcs generate plus or minus signs as follows:

1. Calculate the normalized residual errors between online data *x*(*t*) and its predicted values *x*′(*t*). Let these normalized residual errors be *p*(*t*).
2. When *p*(*t*) deviates from the already calculated *p*′(*t*) by a predetermined extent (called the threshold), then the test to *x*(*t*) is minus (evaluated as faulty), and plus otherwise.

**Figure 3.**
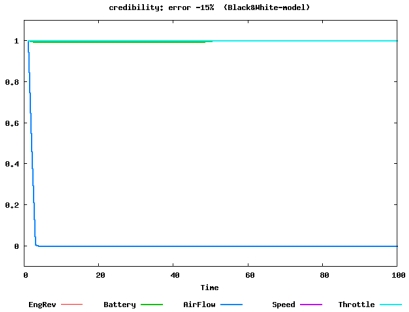
Diagnosis using profiles (parameters and thresholds) calculated from the VAR model when the air flow sensor is faulty. The plotted line shows the time evolution of credibility for the sensor; only the credibility of the faulty sensor becomes 0, hence the diagnosis is successful [6]. © 2006 Springer

The parameters of the VAR model and the thresholds are considered as profiles characterizing the normal state of a specific phase (e.g., cruise phase). Using the profiles, the fault of the air flow sensor is more accurately identified and diagnosis is successful. Figure 3 shows the time evolution of credibility. Only the credibility of the faulty sensor (air flow) becomes 0, hence the diagnosis is successful. It should be noted that the above calculation is done only using normal sensor data.

### 4.3. Training and Tuning: Managing trade-offs

We have demonstrated that the dynamic relational network model can define and generate profiles. A statistical analysis is used for building the network and a time series analysis is used for determining the thresholds for evaluating signs. Only normal sensor data are used for building the network and determining the thresholds.

When the data for abnormal cases are incorporated, the missed-alarm rate for abnormal events is greatly reduced. However, when restricted to normal profiles, there could be many profiles for an event category level. To detect an event accurately (not only in cruise phase but also in idling phase), other normal profiles for idling phase would be required.

## 5. Home Security Sensor Systems Example

**Figure 4.**
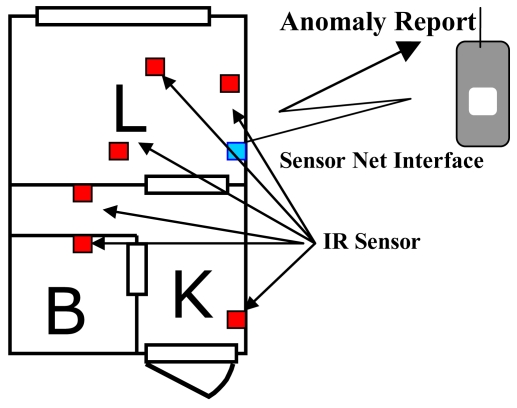
Layout of sensors in a room for the experiment. (K: kitchen, L: living room, B: bathroom).

In this example, multiple homogeneous sensors (infrared sensors) installed in a residence are used for identifying the residents' activities. The sensor systems must not only monitor the residents' activities but also detect events such as housebreaking and a resident collapsing due to a sudden illness. Only normal data (data of the residents' activity) are used for building the dynamic relational network and extracting profiles characterizing the normal data. The focus is on detection of housebreaking, but a resident collapsing is also considered. A statistical method of estimating parameters of the Markov model is used for extracting profiles, and so parameters of the Markov model are used as profiles. The dynamic relational network is built and profiles are extracted off-line using the normal data of the resident's activities. Sensor data are sampled by infrared (IR) sensors installed in rooms in the residence as shown in Figure 4. The detection system processes the data obtained through a sensor net interface.

### 5.1. Modeling: Identifying relations among sensors

**Figure 5.**
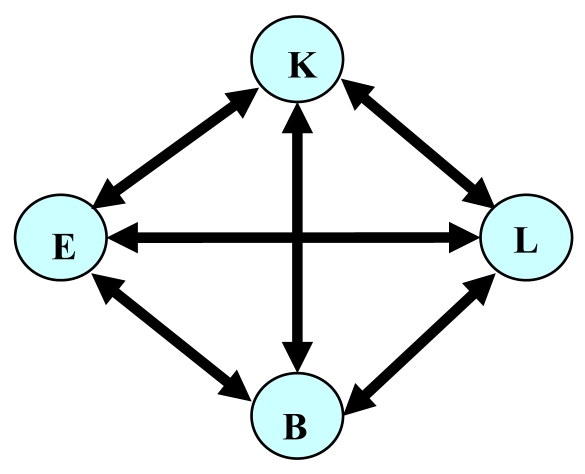
Infrared sensors in each room (E: entrance, K: kitchen, L: living room, B: bathroom) have a probabilistic relation identical to a transition diagram of the Markov model. Each state indicated by a node means the sensor in the room is activated.

We suppose that the sequential activation of sensors installed in each room should have a probabilistic dependency which may be described by the Markov model. For example, if a sensor at the entrance is activated, then a sensor at the kitchen will be activated with a certain probability in a certain period of time. Thus, the sensor activations may be described by a Markov model or *Hidden Markov Model* (HMM) (e.g., [8,9]). Even when restricted to statistical methods, however, there have been many methods such as *Support Vector Machine* (SVM) [10] and particle filter approaches [11]. For human health monitoring, sensors can be attached to the body [12]. Restricted to those related to applying the Markov model to monitoring of human activity by sensors installed in houses, two problems arise: a structural problem and a time-related problem. Even for a single resident, multiple Markov models or even more sophisticated structured Markov models are required, since the resident could have multiple behavior patterns (corresponding to life on a weekday or at the weekend, for example). As for the time-related problem, Markov models have difficulties in handling time, for the first-order Markov model assumes dependency on only one step past; furthermore, Markov models cannot deal with continuous time between activation of a sensor and the subsequent activation of another sensor. We focus on the time-related problem by involving the dynamic relational network. Figure 5 shows a network whose arcs indicate a probabilistic relation identical to a transition diagram of the Markov model.

The activities of a resident are monitored for three months. Since actual abnormal events would rarely occur, virtual anomalies have been set in order to analyze the performance of the system. The following three types of anomalies are presented to the system:

1. Housebreaking from the entrance,
2. Housebreaking from other than the entrance (e.g., from a window), and
3. Resident collapses due to sudden illness.

Among the monitored data, up to five days are used as learning data to train the HMM.; the rest of the data are used to test the detection performance.

### 5.2. Profiling: Identifying events and their characteristic parameters and thresholds

**Figure 6.**
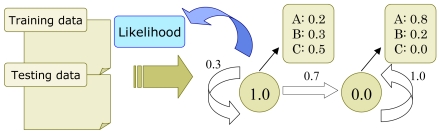
First, parameters of HMM as profiles will be set by training data. Then, test data are given to calculate the likelihood to investigate that the HMM with parameters trained is likely to generate the test data.

The sensor system monitors the resident's usual behavior, extracts normal activities, and updates the normal activity profile. A deviation from the profile can be used as evidence of an anomaly. A collection of parameters of the HMM are used as a profile (Figure 6). The HMM is suited for tasks involving the handling of time series data such as speech recognition and gesture recognition systems [13]. Since the HMM assumes that states are not directly observable, the parameters include output probabilities and initial distribution of probabilities, in addition to state transition probabilities. These parameters are estimated from the data obtained by the sensor system.

The data of the first few days (up to five days) sampled from the sensors monitoring a resident's activity in his/her home are used for estimating the parameters, and the collection of parameters is regarded as the profile of the resident to identify his/her normal life in the home. We call this period of a few days the *training period*, and the data collected during this time *training data*. After the training period, the detection will be carried out by calculating the likelihood that the current data are within the range expected from the normal life, by testing against the profile of normal life (Figure 6).

**Figure 7.**
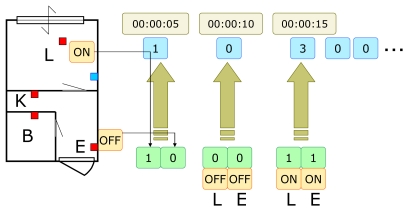
Sensor data codin0g for HMM.

Sensor data must be coded into an input sequence of symbols for the HMM. In the experiment, sensor data are sampled and transformed into a 1 (reacted, or ON) / 0 (not reacted, or OFF) sequence of two bits for every pair of sensors (hence a sequence of four numbers: 0, 1, 2, 3 corresponding to 00, 01, 10, 11 respectively) (Figure 7) every five seconds. To define consistency between sensors, distinct HMMs are used for every pair of sensors. A pair of sensors is consistent if the likelihood computed from the HMM and the current sensor data is more than the threshold, which has been predetermined by the training data and a parameter named sensor reaction range. In this way, a pair of sensors, rather than each sensor, acts as detectors. We can further extend this combinatorial extension by considering triplets and quadruples of sensors, and so on, in which case the relational network would be a hyper graph, or we may even consider higher consistency. This paper does not consider these extensions.

**Figure 8.**
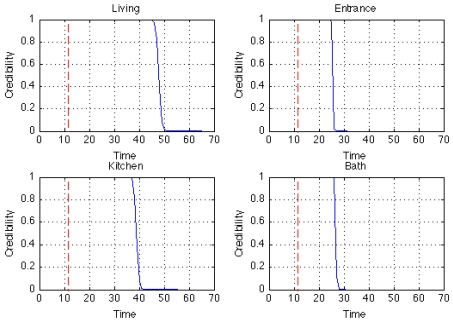
Credibility of each sensor plotted when intrusion from the entrance occurs. The dotted line shows the time when the intrusion started, while the solid line shows the time evolution of credibility for the sensor. Starting from the sensor at the entrance, the credibility of all sensors is lowered because the data do not agree with the profiles of the resident.

With the model and profiles above, the case of intrusion detection from the entrance is tested. Figure 8 plots the time evolution of the credibility of each sensor. Starting from the sensor at the entrance, the credibility of all the sensors is lowered because the data do not agree with the profiles of the resident. In this experiment, the sensor system will issue an alarm when the credibility of any sensor becomes less than 0.5, although the alarm condition could depend on any logical or weighted sum, or even a dynamic pattern of credibilities.

### 5.3. Training and Tuning: Managing trade-offs

**Figure 9.**
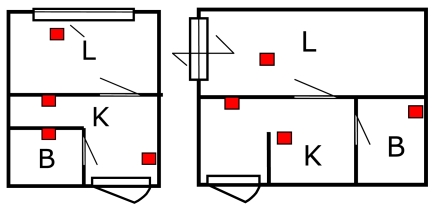
The IR sensor layout in the room of home A (left) and B (right) for the experiment. The IR sensor indicated by a square is installed at each room. The living (L), kitchen (K), bedroom (B), and the entrance are shown.

We conducted the above experiments for two homes having different floor plans (Figure 9) to compare the performances, and thereby investigate and narrow down the factors that affect the performance.

**Figure 10.**
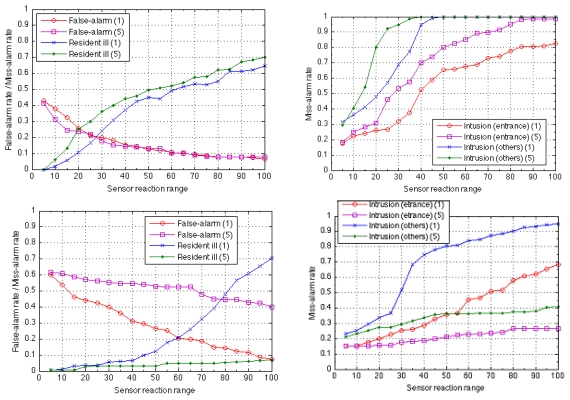
Average rates of false-alarms (left) and missed-alarms (right) of home A (above) and home B (below) when sensor sensitivity (sensor reaction range) is varied. The numbers in parentheses indicate the number of days used for training the system. In the left plots, the missed-alarm rate of the resident collapsing due to sudden illness (indicated by “Resident Ill”) is plotted in addition to the false-alarm rate of intrusion from the entrance.

Figure 10 shows the plots of average rates of false-alarms and missed-alarms for both homes. It can be seen that the performance of the system for both homes is similar, even though the floor plan and hence the sensor layout differ from each other. This means that the system offers adaptability to the sensor layout as long as the number of sensors and coverage of the room are adequately set. In this experiment also, the number of IR sensors is equal (four) and at least one IR sensor is installed in each room: living (L), kitchen (K), bedroom (B), and the entrance.

Among the monitored data, up to five days are used as learning data to train the HMM; the remaining data are used to test the detection performance. The rate of false-alarms in a day (Figure 10, left) as well as the rate of missed-alarms (Figure 10, right) are plotted for various reaction ranges on which the sensitivity depends. When the detection sensitivity decreases by lowering the thresholds for each HMM, the number of false-alarms decreases (Figure 10, left) while the missed-alarm rate increases (Figure 10, left). As expected, this trade-off holds for two data sets from two different homes. The event of the resident collapsing, for which the missed-alarm rate is higher than that for housebreaking, is difficult to detect. In this experiment, the time taken to encode sampling time sensor data into a sequence is five seconds. If the sensor data are sampled more often, this would raise the missed-alarm rate while lowering the false-alarm rate, for this would give more data of normal cases in the training.

**Figure 11.**
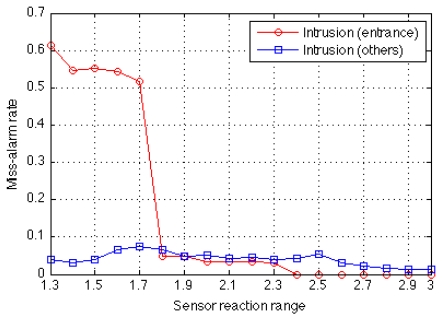
Missed-alarm rate decreases if the profile describing abnormal activity (intrusion from the entrance and from other places) is introduced.

In this example of home security, the missed-alarm rate could be reduced if profiles for specific abnormal events were available. Figure 11 plots the missed-alarm rate when the profile describing abnormal activity (intrusion from the entrance and from other places) is introduced.

It is expected that if activities are monitored more frequently by sampling the data from the sensors in less than five seconds, the missed-alarm rate would be improved. As a future work, the sampling time should be adapted to the environment. The experiments demonstrated that anomaly detection based on adaptive updates of the resident's normal behavior allows not only detection of behavior anomalies but also adaptation of the system to the environment. Here, the environment includes dynamic and diverse patterns of abnormal and normal behavior, and dynamic but periodic living patterns. Reflecting the periodic conditions in the short term such as hours and in the long term such as months and seasons to the profiles would improve the rate of successful detection.

## 6. Conclusions

For sensor systems (as opposed to a single sensor measurement), we first need to expand the measurement to involve multiple and heterogeneous sensors, and to extend the process to involve post-processing of data for improved event detection, identification and prediction. To this end, we introduce models and profiles to be defined and generated by the model.

Even in a simple design problem of single sensor sensitivity, we face a trade-off between false-alarms and missed-alarms. When designing sensor systems involving multiple and heterogeneous sensors, we face a system-level trade-off: if a profile-based approach is adopted, we need not only the profile characterizing the normal state but also its dual: the profile characterizing the abnormal state. Without it, we have to detect anomalies as complementary events (event not matching the normal profile), in which case the missed-alarm rate increases. However, there is an intrinsic asymmetry in the availability of the normal profile and the abnormal profile. Although normal profiles are readily available, abnormal profiles are difficult to obtain. This asymmetry requires systematic synthesis of abnormal profiles, similarly to the one realized by acquired immunity. Adaptation is required, since the changing and diverse environment implies the need to move around on the trade-off curve, or even the validity of the curve itself is questionable. Designing a sensor system involves solving the problem of mapping signals to a model to attain a given mission.
